# Talking About Gene Drive in Uganda: The Need for Science Communication to Underpin Engagement

**DOI:** 10.1177/10755470241234048

**Published:** 2024-03-11

**Authors:** Sarah Hartley, Aleksandra Stelmach, Chris Opesen, George Ladaah Openjuru, Stella Neema

**Affiliations:** 1 University of Exeter, UK; 2 Makerere University, Uganda; 3 Gulu University, Uganda

**Keywords:** Uganda, gene drive mosquitoes, engagement, social justice, science communication

## Abstract

Uganda may host the world’s first field trials of gene drive mosquitoes for malaria control. Global North discourses pre-suppose African publics have access to information about gene drive and are ready to make decisions about its governance. We explore assumptions about the availability of this information in Uganda. We find a paucity of information available combined with a strong desire for information from lay publics. We discuss these findings in the context of Ugandan information infrastructures and political sensitivities to genetic technologies. If Ugandans are to decide about gene drive, they need independent information about the science to underpin engagement.

## Introduction

The development of a radical new biotechnology called gene drive to reduce or eliminate malaria across sub-Saharan Africa has triggered a debate about how to govern it responsibly and who should be included in governance decisions. Target Malaria, an international research consortium, is developing gene drive mosquitoes that will suppress wild mosquito populations by ensuring only male offspring in future generations. Unlike other genetically modified organisms (GMOs), gene drive organisms are designed to spread in wild populations, which means they could cross regional and national boundaries. Uganda, a country with a significant health and economic burden from malaria, may be the first in the world to test gene drive mosquitoes in the wild. While there are significant hopes in Uganda that gene drive could alleviate malaria (Hartley, Smith, et al., 2021), the technology is controversial and it is not yet clear how decisions about whether or not to release gene drive mosquitoes will be made (de Graeff et al., 2022). Despite the uncertainty about governance and decision-making, there is frequent mention in the academic literature and reports about how “Africans will have the ultimate say in when and how these technologies will be used” (Neves & Druml, 2017, p. 3). Assertions that decisions about gene drive were in the publics’ and Africans’ hands have also appeared in the press (Stelmach et al., 2022).

These and similar claims rest on the idea that the decision-making process concerning gene drive should be inclusive, democratic, and underpinned by engagement (Kaebnick, 2021). In gene drive literature, engagement constitutes a key component of gene drive governance (National Academies of Sciences, Engineering, and Medicine [NASEM], 2016). It is enshrined in the gene drive community’s code of ethical conduct (Annas et al., 2021) and principles for gene drive research (Emerson et al., 2017), where it is described as “transparent dialogue [. . .] critical for enabling well-informed public discussion and debate” (Emerson et al., 2017, p. 1135). While science communication understood as provision of information is still considered important for engagement, and for “forming opinions and decision making” (NASEM, 2016), it is no longer the only concern. More broadly, while it is recognized that information is needed for meaningful debate and decision-making, engagement is increasingly in demand (Weingart et al., 2021). In the case of gene drive, engagement is seen as a bedrock on which decision-making processes should be founded. Such engagement would include in these decisions not only local communities hosting field trials, but also stakeholders and broader publics (NASEM, 2016), and it would need to take into account social and environmental justice and procedural fairness (de Graeff et al., 2022). This focus on engagement reflects a normative commitment to the idea of democratic science policy that is present in much of the current public engagement scholarship (Burgess, 2014). It also reflects a shift of attention from communicating science to engaging with publics, emphasizing the notion of societal dialogue or deliberation rather than sharing information about science and technology with various sections of a society (Stilgoe et al., 2014).

Our goal is not to make the case for public engagement in governance decisions about gene drive mosquitoes in Africa. Others have already done this very effectively (Kuzma et al., 2018). Rather, our goal is to shift the focus back on the issue of science communication and its importance in the context of the African countries expected to host field trials, especially in Uganda. Science communication and the provision of information about science underpin engagement and are central to many societal challenges (Horst et al., 2016; Rowe & Frewer, 2005). Arnstein’s (1969) seminal paper introducing a ladder of citizen participation understands engagement in terms of power sharing, but it recognizes the importance of information sharing, which she notes is a crucial step in determining public participation in decision-making. As Arnstein claims: “Informing citizens of their rights, responsibilities, and options can be the most important first step toward legitimate citizen participation.” She also notes that when “information is provided at a late stage in planning, people have little opportunity to influence the program designed ‘for their benefit” (p. 219).

Yet, the value of information sharing and communicating about science is often overlooked, taken for granted, or considered as part of or in relation to engagement (Davies, 2022). Indeed, a common assumption, especially in the Global North context, is that information about science and technology is available to people (Ko, 2016), and that exclusion from science communication is caused for example by “barriers,” such as a lack of interest or motivation on the part of lay publics (Dawson, 2014). There are exceptions, including military research, ecological disasters, or health controversies where the existence and development of technologies are shrouded in secrecy, and where the information about environmental or health damage they cause is withheld by powerful actors (Proctor, 2012; Rappert et al., 2008). A recent literature suggests, however, that information about science and, by extension, opportunities for engagement with science are unequally distributed within and across societies (Besley, 2009; Canfield et al., 2020; Dawson et al., 2022). If science communication, as Medvecky (2018) argues, is about knowledge distribution, then the issues of the access to and sharing of knowledge and information about science have implications not only for engagement but also for ethics and epistemic justice (Priest, 2018). This approach to science communication allows exploration of who has access to information about science and who does not, what it means for information sharing as part of power sharing (Arnstein, 1969), and how structural inequalities can shape deliberation and decision-making (Young, 2001).

We take the issue of science communication and access to information about science as a starting point for our exploration of the case of gene drive in Uganda. As gene drive mosquitoes are designed to spread, there is a need for an analysis of the public communication of this technology that goes beyond communication and engagement activities targeting local communities, and which extends its attention to the national and international level. Davies (2022) stresses the importance of examining the context in which science is (or is not) communicated. As Davies (2022) writes, “Understanding the landscape of a particular issue may well result in identifying pathologies or lacunae within public debate on it [. . .]. Where is there a need for information, particular forms of communication, or engagement or deliberation?” (p. 4). In our study, we explore these issues by drawing on Bauer’s (2002) conceptualization of public spheres of science and technology. We examine who is talking about gene drive in public spheres in Uganda and what information might be needed to enable public debate and decision-making on gene drive. We then contextualize our findings against the background of international discussions about gene drive.

Information about science and technology plays a critical and essential role in engagement. Yet Uganda is information-poor, with low levels of literacy, and relies on political institutions with limited transparency (Conroy-Krutz, 2016). Access to information on recent scientific developments in medicine, science, and technology is extremely limited, with the poorest sections of society unable to access information about advances in health care, agriculture, or the causes of climate change (Brown et al., 2018; Godfrey et al., 2012). Information poverty is particularly acute in Uganda in part because of information illiteracy, lack of resources, governmental censorship and control, established information policies or lack thereof, and internal information infrastructures (Gebremichael & Jackson, 2006, p. 268). Such information poverty might make it difficult for public discussion and for “Africans to have the ultimate say” in decisions about if or how gene drive mosquitoes might be used.

Communication of political and scientific issues is complex in Uganda. Engagement in political decisions is constrained as citizens do not know enough about processes, actors, and issues to participate in (Conroy-Krutz, 2016). We use media analysis, interviews, and focus groups to investigate the state of public communication and the availability of information on gene drive in Uganda in different spheres of public life, including the media, elite, and expert discourses, as well as lay discourses about this technology. Our findings show a strong desire for information from the lay publics, yet there is little public information available, and few people are talking about gene drive. This means that the people most likely to be affected by the technology do not have access to information about it, and most discussions happen among elites with decision-making power. This lack of information is situated in a context where there are weak information infrastructures and a sensitivity among elites to political controversies surrounding gene technologies in Uganda. We argue that if Ugandans are to have a role in decision-making about gene drive, they need access to more information about this technology.

## Preparing for Gene Drive Mosquitoes in Uganda

Gene drive mosquitoes are an emerging biotechnology designed to eliminate or dramatically reduce populations of *Anopheles gambiae*, the primary malaria vector in sub-Saharan Africa. Gene drive involves modifying the mosquito’s genome with a desired trait, for example, so that it produces only male offspring, and then using a drive mechanism to “drive” the genetic change through the whole population. The “drive” ensures the genetic change is passed on to subsequent generations by skewing rules of inheritance. Usually, offspring have a 50% chance of inheriting a particular trait from a parent, but gene drive “biases inheritance” so that the new genetic trait is passed to all offspring. In theory, gene drive mosquitoes could eliminate an entire population. No gene drive organism has yet been tested in the field, but precursor field trials of genetically modified mosquitoes took place in Burkina Faso in 2019 (Yao et al., 2022). These mosquitoes provided no health benefits, but their release was designed to test the regulatory process and community engagement efforts.

Uganda has one of the highest burdens of malaria cases in the world, with 90% to 95% of the population at risk of the disease (Kigozi et al., 2020). Transmissions occur all year round and account for about 35% of all hospital admissions (Yeka et al., 2012). Malaria is also one of the biggest child killers and accounts for about 13% of deaths in children under five in Uganda (D. Roberts & Matthews, 2016). The disease significantly impacts the economy due to loss of workdays and low productivity caused by sickness and constitutes a huge financial burden on the state and individuals. For example, a single episode of malaria costs a family about 3% of their annual income (Republic of Uganda, 2015).

The huge human health and economic costs of malaria make gene drive an extremely attractive tool. Ugandan stakeholders have expressed their hope that the gene drive will eliminate mosquitoes, thereby reducing malaria, decreasing reliance on chemical pesticides and mosquito nets for mosquito control, and increasing economic productivity (Hartley, Smith, et al., 2021). Gene drive technology is being developed by Target Malaria, a research consortium based at Imperial College London and partnered with the Uganda Virus Research Institute in Entebbe. The consortium is funded by the Bill and Melinda Gates Foundation and the Open Philanthropy Project (Hartley et al., 2019).

The first field trials of gene drive mosquitoes are likely to take place in one of Target Malaria’s partner countries, such as Uganda, Burkina Faso, or Ghana, in 5 to 10 years (Scudellari, 2019). This is a significant responsibility and considerable effort is going into preparation of legislation and regulatory frameworks (Hartley et al., 2023). The African Union (AU) and the Economic Community of West African States lead regional efforts to develop regulatory frameworks for gene drive and the AU’s development agency, AUDA-NEPAD, actively supports capacity-building for gene drive regulation and governance (Thizy et al., 2020). In 2020, the AU’s High-Level Panel on Emerging Technologies singled out gene drive mosquitoes as one of three priority technologies to contribute to malaria elimination. However, in Uganda, no legislative framework is currently in place to support field trials, although there have been attempts to introduce new legislation. Most notably, the Genetic Engineering Regulatory Bill was passed in 2018, but the President refused to assent it, citing several ecological and health concerns (M. Duba, 2021). GMOs are contentious in Uganda and the Bill (also called GMO Bill), which is heavily focused on GMOs with little mention of gene drive, has been one of the most debated bills in Uganda’s history (Bendana, 2020).

In 2018, the African Union High-Level Panel on Emerging Technologies called for increased public education and awareness on gene drive, particularly for young people to “prepare them for their future role as decision-makers” (New Partnership for Africa’s Development [NEPAD], 2018, p. 32). More recently, the African Institute for Development Policy (AFIDEP) launched the Platform for Dialogue and Action on Health Technologies in Africa to stimulate balanced and objective debate on gene drive (and other emerging technologies). Its goal is to ensure that African communities, civil society, researchers, and government officials are able to shape discussions about the technology and its governance (T. Duba & Tuor, 2021). In Uganda, AFIDEP partners with the Center for Policy Analysis (CEPA, 2022) to “promote and facilitate meaningful engagement and involvement of Africans in driving conversations and action about the need for transformational tools and technologies” with a focus on gene drive mosquitoes.

On the ground, Target Malaria has been conducting community engagement around the site where the first field trial site is likely to take place, as well as stakeholder engagement with experts, administrators, and policy-makers (Pare Toe et al., 2022; Thizy et al., 2019). This is not an easy task with some commentators noting that “conducting trials of high-end science, and of controversial and potentially non-reversible innovations in settings of poverty and with populations where many have had access to only basic education, is ethically challenging and requires careful public engagement” (Beisel & Ganle, 2019, p. 167). In many ways, the gene drive community of developers and supporters conducts exemplary public engagement in emerging technology development, by committing to knowledge co-production and developing novel engagement practices sensitive to local contexts (de Graeff et al., 2022; Hartley et al., 2019). However, African civil society organizations have critiqued these efforts, arguing that Target Malaria dominates engagement activities in Africa, and pointing to what they perceive to be a lack of independent, unbiased public engagement (African Centre for Biodiversity, 2018). The AU, AUDA-NEPAD, AFIDEP, and CEPA all receive funding from the Bill and Melinda Gates Foundation (BMGF, 2022) which is also Target Malaria’s main funder. The reliance on the same funder for technology development, regulatory development, and public engagement and debate raises legitimate concerns about conflicts of interest and about the degree to which scientific information is unbiased.

## Methods

We adopt an interpretive approach (Yanow, 2000), which presumes that social worlds can be lived in multiple ways. Knowledge is acquired through an interpretation by social actors and is dependent on these actors’ prior knowledge and context. An interpretive analysis emphasizes the meaningfulness of human action and attempts to make sense of the intentions underlying the practical reasoning of actors (Yanow, 2000). Local knowledge is indispensable in uncovering the meaning-making of actors and in explaining the findings.

We seek to understand the state of public communication and availability of information on gene drive in Uganda, the ways in which various actors make sense of the discussions about this technology, and the implications of our findings. We adopt Bauer’s (2002) model of public spheres of science and technology according to which debates about innovations occur in three main arenas: mass media, regulation and policy-making, and everyday conversations and perceptions. We designed a study that combines (a) qualitative content analysis of the media coverage of gene drive in Uganda; (b) interviews with Ugandan expert stakeholders (including regulators and policy-makers); and (c) focus groups with secondary school teachers of arts and humanities and of biology in Uganda. The three sets of data generated through these methods were analyzed and interpreted in the current social and cultural context of Uganda. Three of the authors—Opesen, Openjuru and Neema—have an in-depth knowledge of current issues in Uganda and their expertise contributed to data analysis and interpretation. In addition, Opesen led on sampling and recruitment of participants and conducted interviews and focus groups in Uganda in a culturally sensitive fashion. All authors have researched gene drive in Uganda for several years and drew on this broader contextual knowledge.

We used the media database Factiva to identify articles for the qualitative content analysis of media coverage (Bauer, 2000). We searched Factiva for articles published in Uganda between January 1, 2015, the time when the gene drive technology started to emerge, and December 31, 2021. We searched all Ugandan media outlets available on Factiva with the search term “gene drive.” Our search has limitations, as Factiva only records six major English-language media outlets in Uganda: *Daily Monitor, New Vision, The Observer, The Independent, Sunrise*, and *The Red Pepper*. English is the official language used in Ugandan grade schools and by most newspapers (BBC Media Action, 2012). Supplementing Factiva searches with regional newspapers in local languages in the sample proved difficult, given that this study was conducted during the COVID-19 crisis. The pandemic exacerbated the already fragile state of several regional newspapers in Uganda and led to their permanent closure (Muhindo, 2020). Lockdowns also left schools and public research archives closed with a few services in the category of essentials left operational.

To contextualize our sample and the findings of the media analysis, we surveyed academic literature on the landscape of science communication and science journalism in Uganda. We also drew on the lived experience and tacit knowledge of co-authors of this study who are social scientists based in Uganda, and who have followed public debates about genetic technologies in this country for the last decade.

We conducted qualitative in-depth interviews using purposive sampling (Robinson, 2014) to ensure that the sample includes the most relevant stakeholders and reflects the local context and a diversity of perspectives. Although the sample is small, it includes some key Ugandan stakeholders in this field. We recruited 10 participants (P1-10) for the interviews: gene drive developers who are natural scientists working on gene drive technology in Uganda (two participants); natural scientists with expertise in molecular biology and entomology who were not directly involved in gene drive research but observed and were able to assess its progress (three participants); a social scientist with expertise in societal implications of gene drive technology (one participant); a science communicator with expertise on the issues surrounding the communication of gene drive (one participant); a representative of non-governmental organizations scrutinizing the development of gene drive in Uganda (one participant); an expert in legal and regulatory issues (one); and a representative of local government (District Health Officer) from an area with high malaria prevalence and with some knowledge of gene drive (one participant). We asked participants about communication activities related to gene drive that were happening in regulatory and expert circles as well as in public sphere in Uganda, and we probed questions of possible strategies that could or should be used when communicating about this technology. We also discussed the wider social and political context in which gene drive was being developed in Uganda, as well as the prospects of its regulatory approval, social acceptance, and potential deployment.

We conducted two focus groups with secondary school teachers: one with six arts and humanities teachers (P11-16), and another one with six biology teachers (P17-22). Teachers are laypeople regarding gene drive but they are experts in conveying complex information in an accessible fashion. They play a key educational and communication role in Uganda where about 50% of the population is below the age of 15 years (Uganda Bureau of Statistics, 2016). We recruited teachers from state schools in one of the rural regions of Uganda with a high prevalence of malaria.

Focus groups and interviews were conducted in September and October 2020 in compliance with COVID-19 social distancing rules in place at the time. We conducted focus groups in person in a culturally sensitive way, sitting outside underneath a large tree in the community. However, due to COVID-19 restrictions on the number of people who could gather, we limited the number of participants in each group to six people, with participants sitting two meters apart. We were not able to conduct focus groups online given that our participants lived in a rural area of Uganda where internet access was not always possible. Focus groups and interviews were digitally recorded and transcribed. Transcripts were anonymized and shared between the authors. Data analysis was iterative and designed to identify key concerns and discourses and highlight the cultural context underpinning these themes. The findings were then situated in the context of the literature on public communication and engagement with emerging technologies.

## Public Communication of Gene Drive in Uganda

### Talking About Gene Drive in the Media

The media landscape and channels through which people get political information in Uganda is complex and fragmented. The most common sources of political information are: friends (87%), radio (86%), posters (78%), family (77%), parties and rallies (76%), candidates themselves (74%), community meetings (56%), religious leaders (45%), newspapers (43%), chiefs (33%), civic-education campaigns (33%), television (30%), and the internet (9%) (Conroy-Krutz, 2016). Although the radio is the key mass media in disseminating information, there are no digital archives of Ugandan radio shows available for research. We therefore chose to search for newspaper articles on gene drive which are more easily accessible. While newspapers are not the main source of information in Uganda, they are often the first link in the chain of dissemination, as press articles are picked up by local radio stations that reach wider, often rural, audiences. Our search indicated that news reporting on gene drive was extremely limited. According to Factiva, gene drive was covered only by two mainstream newspapers, *New Vision* and *Daily Monitor.* Both outlets are national English-language newspapers with the largest circulation, based in Kampala and widely read by urban populations, but with ambitions to reach wider publics. *Daily Monitor* has an objective to increase its readership in rural areas as well (Wojczewski et al., 2015), while *New Vision* newspaper is part of the New Vision Group (n.d.), a state-owned multimedia conglomerate that owns several newspapers, radio, and TV stations publishing and broadcasting in local languages in Uganda (for an overview of the Ugandan media landscape see BBC Media Action (2012, 2019)).

Given their dominant status in the Ugandan media landscape, *New Vision* and *Daily Monitor* are better resourced than local media outlets. However, over the period of 2015 to 2021, both newspapers published only five articles on gene drive, four of which (Dyer, 2015, 2016; SyndiGate, 2016, 2018) were reprints from the international press. Only one article (Abet, 2021) was written by a Ugandan journalist, drew on local sources, and presented a debate on gene drive among Ugandan scientists.

We asked our study participants—experts (including a policy-maker, an expert in communication and academics following the media coverage), stakeholders, and teachers—about their experience of the media coverage of gene drive. They confirmed that in their experience there was almost no gene drive reporting in mainstream Ugandan newspapers. Similarly, they had never seen news on gene drive on television, though a few stakeholders and teachers remembered coming across some information on this topic on local radio stations. Participants offered a range of interpretations of why there was a gap in gene drive reporting. The most frequent explanation pertained to issues with science journalism in Uganda. According to scientists and academics interviewed for our study, not only was there no tradition of science journalism in Uganda, but working journalists were not trained on how to interact with scientists and policy-makers, how to write science stories, and how to cover gene drive. Given the polarized views on biotechnology in Uganda, a journalist willing to report on gene drive would, as one participant claimed, “stake his life or his work” and could be perceived as corrupt or “bought” by an interest group (P5). In addition, the novelty of gene drive technology meant it was perceived as a “mysterious thing” and difficult to understand (P10), as well as “threatening” and “potentially dangerous” (P5). This made gene drive a topic to be avoided and meant that the coverage could be biased as it was “easier to criticise than go for it” (P5). Journalists were also perceived as interested primarily in covering ad hoc issues of immediate public relevance. As one participant noted, “the media seek for stories of immediate interest, and not about issues or products that will be developed in future” (P10), which meant that it was particularly difficult to engage the media in the topic of gene drive.

By contrast, rather than concentrating on the gaps in science journalism, a few stakeholders, especially experts in communication and policy making, linked the lack of media coverage of gene drive to a lack of communication strategy alongside a lack of proactive engagement with the media on the part of elites. As one stakeholder pointed out: “So it’s not yet in the media, it is just at the level of the regulators and the scientists at the moment, and the scientists have agreed to put a team together to inform the communication” (P3). The lack of media coverage was thus attributed to a lack of concerted efforts of experts to communicate, the issue which we discuss in the next section. Overall, both stakeholders and teachers agreed that there was hardly any reliable information about gene drive in the public realm which in their view resulted in a very low public awareness of gene drive in Uganda.

### Experts Talking About Gene Drive

Although major newspapers are talking very little about gene drive in Uganda, lively conversations about this technology are taking place among some Ugandan expert stakeholders. Stakeholders interviewed for our study identified several organizations leading conversations which they thought were communicating effectively about gene drive in Uganda. These included Target Malaria, Uganda Virus Research Institute, National Agricultural Research Organization, Makerere University College of Agricultural and Environmental Sciences, Malaria Consortium, National Council of Science and Technology, and the Ministry of Science and Technology. However, stakeholders acknowledged that this communication happened mainly in a limited number of expert circles with other experts. As one participant stated, “so far the only people I talk to are fairly educated people” and conversations about gene drive were happening “among a small group of the educated elite” (P5). Participants also identified several barriers to communicating gene drive to wider publics.

Politicization of the GMO debate featured prominently in the interviews with stakeholders, and it was singled out as a significant challenge to communicating about gene drive. As one expert put it:

once you talk about gene drive, it is automatically associated with genetic modification and that is unfinished business. In Uganda, for example, that legislation on genetic modification has been on and off for almost ten years. Parliament passes it and the president, at one time, had even passed it, then he recalled it, then they passed it, I think right now it is still in limbo. Yes, it’s an impassioned debate; there are many people on one side who are very much in favour of gene drive and there are some who are totally against, and both of them have their genuine concerns. So definitely it’s not an easy topic to talk about (P5).

These communication difficulties were compounded by political concerns that gene drive could be viewed as a colonial project of sorts—with the technology initially developed in the United Kingdom and developed and promoted for deployment in a country with a colonial past. The novelty of gene drive technology was also seen as a barrier to communication. Given that the technology was still in development and had not been tested in the wild, it was not clear what experts could reliably tell the lay public about it. As one scientist noted: “I think that’s the gap we currently have with our training. We talk about theory but in terms of applicability we don’t have much to show, so it becomes very hard to convince anyone that it works” (P8).

Some participants thought that communication was fraught with difficulties, especially given that at this early stage, it was easier to make mistakes. For this reason, a policy-maker felt that the communication strategy had to be developed carefully in order not to stir opposition to gene drive: “if there’s communication, if someone puts in a wrong article people will ask questions which would throw many people off balance” (P3). A cautious approach to communication was needed not least given that, according to some participants, there was a widespread lack of understanding of how biology worked and that the hostility toward GMOs could taint public perception of gene drive. In addition, experts believed that lay publics would not want to learn about gene drive, as the topic was “not sexy,” and people were interested mainly “in entertainment and goods to purchase” (P10). In this context, a policy-maker thought it was right that discussions were being held only among small circles of experts and decision-makers—this was supposed to be the first step in communication which should be guided by elite input:

So mainly gene drive discussion is at the regulatory level and at the scientific level, it has not gone into the general public yet; why? Because the team has yet to develop the methods of how to go to the public to discuss it; and you cannot just go to the public and just come up and talk to them about gene drive. So they are doing the right thing; first of all to keep the discussion at the higher level of the policymakers, and then the policymakers will guide and add more desirable input that will help in the strategies to communicate better and to introduce the technology safely (P3).

At the same time, several experts thought communicating benefits was most likely to mobilize public support for gene drive. Generating “acceptability” (P7) was viewed as a desirable outcome of communication which could be achieved by using a simple language adapted to the lay public’s needs. As one scientist put it, “if you approach it from that angle, [. . .] you will get people [to] say they will be your advocates” (P7). Stressing health and financial benefits was viewed as effective in garnering future support. In the words of one scientist, “if I’m able to reduce mosquitoes that means I have more money in my pocket and I can use that money to buy something for my family” (P8). A policy-maker argued that communicating health benefits was particularly important:

You’ll not be bitten [by mosquitoes] and moreover that mosquito is the one that bites most at night so you’ll sleep soundly; you will not be having to pray to go to hospital to get malaria medicines. So they [the public] will see it in terms of benefit rather than some adventure in science (P3).

Not everyone agreed with this view. A social scientist argued that an excessive focus on positive messaging could amount to “indoctrination” about the benefits of gene drive (P5). A communicator interviewed for our study also felt that communication should be less about persuading people to accept the technology, and more about “dialoguing” (P4) with them and working to develop tools that would help them understand the technology. However, these were exceptions. Overall, experts thought future communication should help people “embrace” the technology (P3), and a carefully designed dissemination plan could help achieve this goal.

While experts refrained from talking about gene drive in public, they had well-formed opinions on who should be involved in the future public communication campaign. Experts identified a range of actors who would need to be enrolled, starting with health authorities and professionals, especially those who had expertise in communication, malaria control, and treatments and who enjoyed trust in local communities. Religious leaders, alongside community leaders, were also seen as key actors able to attract the attention of people in future communication activities. There was some disagreement among experts on whether politicians should be involved in the information campaign. While some participants feared politicians could potentially provide people with biased information about the technology, they were also considered crucial players in the process of dissemination, as well as regulation and decision-making.

In these discussions, schools and teachers emerged as key actors who, according to virtually all the interviewed experts, could successfully communicate gene drive to lay publics. Schools were considered a suitable institution for this purpose as it is in schools that “the right perceptions” and attitudes are formed, alongside “the right thinking mind-set” (P3). Schools alongside universities were also identified as a place where people would be provided with correct information about gene drive. In addition, educating youth would potentially result in educating their parents, as the information about gene drive would be passed on in families. Overall, schools and teachers were seen as a key ingredient necessary to deploy the information strategy on the ground. However, it was not clear when such information campaigns would take place, and there were no plans on how to train teachers or incentivize them to take part. For the time being, no large-scale information or education activities were taking place and experts had no information about the state of public knowledge or attitudes to gene drive in Uganda.

### Teachers (not yet) Talking About Gene Drive

While experts envisioned a prominent role for teachers in communicating about gene drive, the majority of teachers taking part in our study were unaware that this technology was being developed for potential future use in Uganda. They also struggled to identify any official sources—media, authorities, or other information channels—that could provide them with information about it.

There are many channels by which political and scientific information is disseminated in Uganda. Informal channels such as family and friends are important routes (Conroy-Krutz, 2016), as well as more traditional ways of dissemination in communities, for example, drumming, performances, and cultural and religious events (for an in-depth account of the science communication landscape in Uganda, see Lukanda, 2020). In the experience of the teachers taking part in our study, none of these channels was disseminating information about gene drive, and the technology was not discussed by anyone they knew or interacted with.

There was an exception, as two biology teachers had heard about gene drive before focus groups took place. They had found out about the technology by chance and tried (with limited success) to access more information about it. Two arts and humanities teachers had heard about GM mosquitoes from friends and on the radio but were not able to explain what they were or to relate it to gene drive. Given no prior exposure to the concept of gene drive, most participants had not made up their mind about this technology and formed their opinions about it based on the information emerging from the focus group interactions.

Even without prior specialist knowledge and access to information on gene drive, teachers were able to engage in a conversation about what they saw as potential benefits and risks of this technology. The recurrent theme was the hope that gene drive could help reduce or eliminate malaria, thus improving health and finances of many Ugandans. According to one participant, “when you get rid of malaria, our pocket will also be better because the money you spend on [malaria] treatment will now be put in another area” (P11). Some even expressed the heightened hope that gene drive will deliver for them “a community free from malaria” (P14).

Alongside these hopeful statements, some teachers pointed out the potential risks of gene drive. Even without prior knowledge about gene drive, teachers identified potential negative and unintended consequences of this technology consistent with those identified by another group of expert Ugandan stakeholders (Hartley, Smith, et al., 2021). This included the risk that gene drive mosquitoes might carry malaria parasites causing even more serious illness in humans; that the efficiency of the technology would wane over time as mosquitoes would develop resistance, as has been the case with resistance to spraying with pesticides; and the risk that the introduction of gene drive mosquitoes could adversely affect other species and the ecological balance. While hopeful about gene drive’s potential, teachers also remained level-headed about this technology and articulated a strong need for more easily accessible information. In their view, authorities, medical professionals as well as community and religious leaders should be leading the communication campaign, although some mentioned that teachers should also be included.

Similarly to experts, teachers identified politicization of GMOs in Uganda as a major challenge to communication, alongside the opposition to biotechnology among various groups such as anti-GM organizations and religious leaders. In their view pharmaceutical companies could also be critical of gene drive as they were set to sustain financial losses in sales of anti-malarial drugs and treatments should the technology prove successful. Such criticisms and anti-GM sentiments could negatively influence future public attitudes to gene drive.

In contrast to experts who viewed the lack of public interest and gaps in education as a potentially crucial challenge to communicating gene drive, teachers felt that this topic would engage people as it spoke to their everyday concerns and lived experiences. They were also cautiously optimistic that it would be possible to talk about gene drive in a simple language, including local languages. Unlike experts who emphasized the barriers to communication rooted in the state of science journalism and public attitudes to GMOs in Uganda, teachers thought that an unmet need to provide information about gene drive to lay people was the biggest barrier to communication and debate about this technology in Uganda.

## Discussion

Our study examined how gene drive is being talked about in Uganda and by whom. It suggests there is little information available in the public sphere. Elites responsible for devising communication strategies and authorizing information campaigns are reluctant to communicate given the political controversies surrounding gene technologies in Uganda. The paucity of information disseminated to the wider public is compounded by structural inequalities and weak information infrastructures, which means that lay publics potentially affected by gene drive do not have access to information about this technology.

The Ugandan context is important. There are significant structural barriers to reporting on gene drive in Uganda. Ugandan media houses are small and under-resourced, and there is no tradition of science reporting, which means that science news is often covered by freelancers without adequate training and support (Lukanda, 2019, 2020). Media outlets often do not have dedicated science desks, and science journalists struggle to access scientific news and sources of reliable information (Appiah et al., 2015). Interactions between journalists and scientists are strained, as both sides lack skills in how to communicate and collaborate effectively, with few incentives on how to overcome these barriers (Kaye et al., 2011). As a result, there is an excessive reliance on foreign sources of information, especially from Europe and North America, and a lack of critical reporting of scientific issues (Nguyen & Tran, 2019). These weak information infrastructures mean media coverage of gene drive in major Ugandan newspapers is extremely limited. This stands in stark contrast to reporting in the United States, United Kingdom, and Australian press where gene drive, particularly gene drive mosquitoes in sub-Saharan Africa, has received substantial coverage by major newspapers (Stelmach et al., 2022).

In addition, social and political tensions surrounding GM organisms make gene drive a sensitive topic that scientists and journalists alike may be reluctant to discuss in the media. The politicization of the GMOs debate, the grip of politicians on many science-related issues, and potential backlash from anti-GM activists may have “pushed scientists into silence” (Lukanda, 2020, p. 925), while journalists risk their safety if they write about politically contentious issues (BBC Media Action, 2019). Furthermore, media outlets attempt to represent the views of various stakeholders, which can create “a false balance between the pro- and the anti-GMO activists” (Lukanda, 2019, p. 652). As a result, reporting on biotechnology lacks depth, and it tends to be influenced by the views of stakeholders and by debates in other countries (Lukanda, 2018). For example, Schnurr (2013) has argued that media coverage was driven by well-designed campaigns orchestrated by powerful proponents of biotechnology. Given these challenges, and the difficulties accessing information about science, there was a risk that, as Lukanda (2019) argued, discussions about GMOs would remain the preserve of elites in Uganda.

No public communication strategy is yet in place to inform lay publics about gene drive in Uganda. Political sensitivities to the GM debate in Uganda as well as the novelty of gene drive means that experts are reluctant to talk about gene drive in public. Gaps in communication training as well as fears that mistakes in communication at this early stage could jeopardize the technology also contribute to the delaying of a public communication campaign. These fears are accompanied by beliefs that a lack of interest and low education levels among publics made early communication difficult or even unnecessary. However, these beliefs conflict with our findings which suggest lay publics, such as teachers, have a thirst for independent information about gene drive mosquitoes and are capable of understanding the science and its implications. They felt Uganda’s GMO controversy was a rationale for more information on gene drive, not less, and did not understand why elites limited their access to such information.

Decision-making on gene drive will require an informed citizenry, knowledgeable about the science of gene drive and its implications, including how it works, how it could be used, and what will happen next. This will be crucial for governance and public engagement in political decisions on whether or not to release gene drive organisms. Publics will need independent, accessible, and thorough information to enable them to fully participate in decision-making processes (Burgess et al., 2018). The transboundary nature of this technology places increased emphasis and importance on public communication and broad engagement in decisions about trial releases and potential deployment as those affected will not just be close to the trial site but may include all citizens and even those in neighboring countries. Yet, the focus of much of the activities around gene drive in Africa has been on community engagement around the insectaries to gain community consent for field trials and for post-release monitoring (Hartley et al., 2019; A. J. Roberts & Thizy, 2022). However, a large-scale release of a gene drive mosquito will require societal-level decisions about whether or not to support the release of gene drive mosquitoes in trials and/or eventual deployment. This will involve citizens and not just communities in the trial sites (Bartumeus et al., 2019). Broader communication about the technology is even more important given that gene drive mosquitoes are designed to spread and individual consent will be impossible (George et al., 2019).

Independent information provision may be more difficult in Uganda given existing power imbalances and the influence of Global North actors in this debate. The Bill and Melinda Gates Foundation (BMGF) is the major funder of Target Malaria’s gene drive technology, and there are no other gene drive developers in Uganda. The BMGF has an explicit aim to shape policy and regulatory frameworks and public debate in sub-Saharan Africa in ways that support the development, testing, and deployment of gene drive mosquitoes (Hartley, Smith, et al., 2021). Target Malaria, the African Union’s High-Level Panel on Emerging Technologies, the African Institute for Development Policy, and the Center for Policy Analysis are powerful and well-resourced actors, all funded by the BMGF to promote gene drive mosquitoes, and there are very few alternative communication about this technology in Uganda. Indeed, in the development of gene drive mosquitoes in Mali, Hartley, Ledingham, et al. (2021) found the BMGF’s role in shaping information provision and funding efforts to promote gene drive was often masked behind engagement practices. In Uganda, such a situation may result in a lack of independent information and the potential for conflict of interest in science communication. This presents a very real concern which so far has received little interrogation. Clarity is needed on the purpose of, or motivations for engagement and the distinction between engagement to support project goals and engagement that is for democratic decision-making.

In Africa, information sharing will be critical for supporting and enabling public engagement with gene drive, yet empirical research on how gene drive is being talked about in African countries is scant (but see Chemonges Wanyama et al., 2021). Global North discourses about potential field trials and deployment of gene drive mosquitoes in Africa mobilize the African publics as scientifically informed citizens able to debate and decide about this technology. Our findings suggest that this rhetoric of “Africans deciding” is unlikely in Uganda where the lack of information and communication points to a different reality in which large parts of Ugandan society feature as “non-publics” (Varughese, 2012) excluded from access to information and deliberation. A more likely scenario is that *some* Africans will decide. However, the healthy and vibrant communication ecosystem, where gene drive was taught in schools and discussed throughout society, led by key community members, envisaged by our participants may still be possible. Ledingham et al. (2023) have highlighted the need for independently funded social sciences for this vision to happen (Ledingham et al., 2023). But, barriers exist, including weak information infrastructures, potential public opposition, political controversies surrounding GMOs, and conflicts of interest, yet the decision of the elites not to talk about gene drive in the current political context may present the biggest barrier.

## Conclusion

Over the last two decades or so the concept of public engagement with science and technology has become a defining feature of science policy rhetoric in the Global North and in some Global South countries (Weingart et al., 2021). This has coincided with a global agenda promoted by governments, universities, and scientific institutions “to expand and intensify science communication” and provision of science-related information to the public (Weingart, 2022, p. 288). Much of the literature on public communication of and engagement with science and emerging technologies in the Global North context explores the issue of how best to communicate and engage with lay publics, including how to reach audiences deemed reluctant, disinterested, or hard to engage (for a critique, see Dawson, 2014). Our study explores the availability of information on gene drive in Uganda in a context where reluctance, non-engagement, and non-communication characterize not the public but those in charge of developing, regulating, and informing about an emerging technology. This approach to communication occurs in the context of a communication infrastructure that lacks capacity and transparency. Our findings highlight the imbalances in power, especially regarding the access to information as a pre-requisite to citizen participation in decision-making processes (Arnstein, 1969) and they illustrate the ways in which structural inequalities can bias the deliberation and decision-making “towards more powerful agents” (Young, 2001, p. 671). Power imbalances highlight the inequities and exclusion underpinning the politics and practice of science communication in Global South contexts such as Uganda. Uganda is heavily impacted by malaria and there are ethical justifications for conducting field trials in countries where the potential benefits can be felt by those who need them (Bonsall, 2016). However, our study highlights a need for independent science communication in advance of engagement in the governance of emerging technologies in the Global South, not to gain public support or build trust, but to enable informed decision-making, otherwise the idea that “Africans will decide” will remain elusive.
